# From complex to simple: myogenesis in an aplacophoran mollusk reveals key traits in aculiferan evolution

**DOI:** 10.1186/s12862-015-0467-1

**Published:** 2015-09-18

**Authors:** Maik Scherholz, Emanuel Redl, Tim Wollesen, Christiane Todt, Andreas Wanninger

**Affiliations:** Department of Integrative Zoology, Faculty of Life Sciences, University of Vienna, Althanstraße 14, 1090 Vienna, Austria; University Museum of Bergen, University of Bergen, Allégaten 41, 5007 Bergen, Norway

## Abstract

**Background:**

Recent studies suggest a bifurcation at the base of Mollusca, resulting in the primarily single-shelled Conchifera (Bivalvia, Gastropoda, Scaphopoda, Monoplacophora, Cephalopoda) and the spicule-bearing Aculifera (Polyplacophora, Neomeniomorpha, Chaetodermomorpha). A recent study revealed a complex larval musculature exclusively shared by Neomeniomorpha and Polyplacophora, supporting a close relationship of both taxa. However, the ontogenetic transition from the complex larval to the simple adult neomeniomorph musculature, which mainly consists of a three-layered body-wall musculature and serially iterated dorsoventral muscles, remains unknown. To close this gap in knowledge, we studied remodeling of the larval musculature during metamorphosis in the neomeniomorph *Wirenia argentea*. A comparative analysis with a novel data set of a polyplacophoran, *Leptochiton asellus*, allows us to infer the morphology of the last common ancestor of Aculifera and the evolution of its subclades therefrom.

**Results:**

The complex larval musculature of *Wirenia argentea* persists through metamorphosis and becomes modified to form two of the three muscle layers of the adult body wall. The innermost longitudinal layer of the three-layered body wall musculature is generated by transformation and expansion of distinct larval longitudinal muscle bundles. The larval ventrolateral muscle strands are remodeled and eventually become the most ventral part of the adult longitudinal layer of the body wall musculature. The paired larval enrolling muscle forms the lateral parts and the former rectus muscle is destined to become the most dorsal part of the longitudinal layer of the body wall musculature. The transient ventromedian muscle is lost during postmetamorphic development.

**Conclusions:**

Postmetamorphic remodeling in *W. argentea* supports the hypothesis of a complex myoanatomy rather than a three-layered body wall musculature at the base of Aculifera, and thus argues against homology of the body wall musculature of adult Neomeniomorpha and other potential molluscan sister groups. Our data show that the neomeniomorph body wall musculature is a derived condition and not an aculiferan or molluscan plesiomorphy.

## Background

**Fig. 1:**
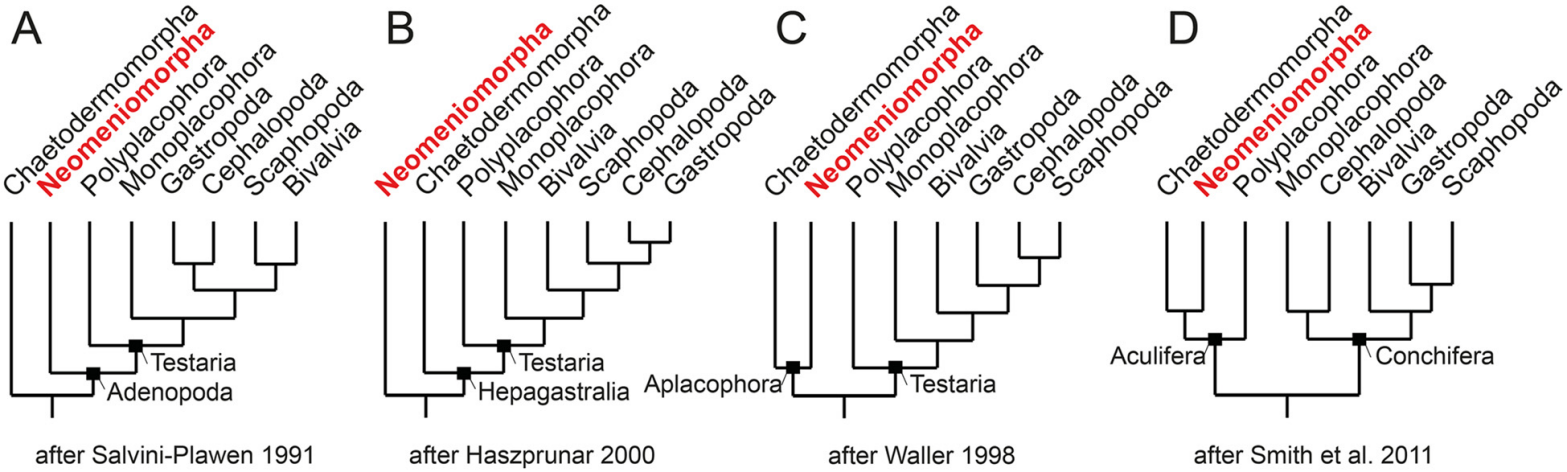
Summary of leading hypotheses of molluscan phylogeny. **a** Adenopoda hypothesis placing the aplacophoran clade Chaetodermomorpha as first offshoot and sister group to the remaining molluscan taxa (Neomeniomorpha + Testaria = Adenopoda) (after [39]). **b** Hepagastralia hypothesis placing the aplacophoran clade Neomeniomorpha as first offshoot and sister group to the remaining molluscan taxa (Chaetodermomorpha + Testaria = Hepagastralia) (after [36]). **c** Testaria hypothesis placing the shell-less Aplacophora (= Neomeniomorpha + Chaetodermomorpha) as sister group to all primarily shell-bearing molluscan taxa (Testaria) (after [47]). **d** Recently supported Aculifera hypothesis showing basal bifurcation by placing Aculifera (= Aplacophora + Polyplacophora) as sister group to all primarily single-shelled mollusks (Conchifera) (after [5])

The extraordinary species-rich phylum Mollusca displays a vast diversity in morphology and has traditionally been subdivided into eight extant class-level taxa. Besides commonly known taxa such as the Bivalvia (clams and mussels), Gastropoda (snails and slugs), and Cephalopoda (octopuses and squids), Mollusca comprises also lesser-known groups: the limpet-like Monoplacophora (Tryblidia), the tusk-shelled Scaphopoda, the eight-shelled Polyplacophora (chitons), and the vermiform, spicule-bearing aplacophoran clades Neomeniomorpha (Solenogastres) and Chaetodermomorpha (Caudofoveata). The effort to reconstruct the evolutionary interrelationships of these eight major lineages of Mollusca has resulted in several competing phylogenetic hypotheses, but to date none of the interrelationships could be unambiguously resolved for the above-mentioned class-level taxa (Fig. 1; see also [1]). The major hypotheses confirm the monophyly of Mollusca and Conchifera, but differ extensively in the position of the respective subgroups to each other and in particular in determining the most basal offshoot of the molluscan tree, thus still rendering the molluscan evolutionary origin enigmatic (Fig. 1).

The morphological diversity of the Mollusca and the diverging phylogenetic hypotheses have led to different conclusions when reconstructing the hypothetical last common molluscan ancestor. A recent hypothesis proposes a vermiform and shell-less but spicule-bearing neomeniomorph-like ancestor [2]. In this scenario, the typical three-layered body wall musculature of recent Neomeniomorpha, consisting of outer ring, intermediate oblique, and inner longitudinal fibers, was considered to be a basal molluscan character [3].

Most recent phylogenomic and mitogenomic studies suggest a molluscan phylogeny with high support values for the monophyletic clades Aculifera (Polyplacophora + Aplacophora) and Conchifera, respectively (Fig. 1d) [4–6]. Aside from molecular analyses the close relationship of Aplacophora and Polyplacophora was recently supported by the description of the fossils *Kulindroplax perissokomos* [7] and *Phthipodochiton thraivensis* [8], both exhibiting a combination of aplacophoran and polyplacophoran characters. Moreover, recent ontogenetic data of the neomeniomorph *Wirenia argentea* suggest that aplacophoran mollusks evolved from ancestors with polyplacophoran-like features [9]. Nevertheless, the basal aculiferan-conchiferan dichotomy raises obvious issues concerning molluscan plesiomorphic character states. In particular, the myoanatomy of the last common molluscan ancestor and the associated question whether the body of this molluscan stem species was either covered by spicules or shell(s) remains unresolved. To this end, investigations of polyplacophoran and aplacophoran ontogeny in an evolutionary framework may allow for conclusions concerning ancestral features of at least the aculiferan lineage within Mollusca.

The Neomeniomorpha develop via a so-called pericalymma larva, a free-swimming lecithotrophic trochophore-like larva with a preoral apical cap (calymma) and a posterior trunk [10–12]. The pericalymma larva features common trochophore characters such as an apical tuft, a locomotory preoral prototroch, and a posterior telotroch. Late larval stages of Neomeniomorpha exhibit a ventro-longitudinal ciliary band at the posterior trunk, which marks the developing creeping sole, and a complex muscle system. The neomeniomorph larval myoanatomy highly resembles that of polyplacophorans and is composed of paired enrolling and ventrolateral muscles, a single ventromedian and a rectus muscle, as well as a seven-fold arrangement of dorsoventral muscles [9]. In contrast, the adult neomeniomorph myoanatomy contains mainly a simple three-layered body wall musculature similar to other vermiform invertebrates, which is completed by multiple, serially arranged outer and inner sets of dorsoventral muscles. While the larval complexity suggests that the Neomeniomorpha are secondarily simplified and presumably emanated from an aculiferan ancestor with polyplacophoran-like musculature, it appears still enigmatic how exactly the rather simple myoanatomy of the vermiform adult neomeniomorphs derives from the complex larval muscle system. Accordingly, we investigated late myogenesis in the neomeniomorph *Wirenia argentea* and reconstructed in detail the remodeling of the somatic musculature after metamorphosis. We here compare our data to novel findings on polyplacophoran myogenesis. In doing so, we test hypotheses concerning the homology of key polyplacophoran muscular elements, including the rectus and the enrolling muscles, with their neomeniomorph counterparts and assess the developmental and evolutionary origins of the postmetamorphic neomeniomorph body wall musculature from its recently described highly complex larval precursors (cf. [13, 14]). This enables us to draw a clearer picture of the muscular toolkit of the last common aculiferan – and potentially also molluscan – ancestor.

## Methods

### Animal cultures

Adult *Wirenia argentea* Odhner, 1921 were collected with a hyperbenthic sled on muddy plain seafloor in Hauglandsosen (190–226 m depth) and Hjeltefjorden (227–312 m depth) (Bergen, Norway) during January - March 2012, as well as from November 2012 to January 2013. Rearing was conducted at the marine animal facilities of the Department of Biology, University of Bergen. Adults, embryos, and larvae were cultivated at 7 °C in Millipore-filtered and UV-treated seawater following the detailed description of Redl et al. [15]. Staging of investigated specimens was based on morphological aspects due to chronologically heterogeneous late development. Thus, the oldest stage prior to completion of metamorphosis (16–20 days post hatching, dph), herein referred to as “metamorphosing larva”, still exhibits at least vestiges of the larval apical cap and a posterior telotroch. Vermiform individuals shortly after completion of metamorphosis lack these larval features and were categorized as “early juveniles”. The oldest individual examined was 19–21 dph. In addition, adult specimens collected from the natural habitat were investigated.

Adult *Leptochiton asellus* (Gmelin, 1791) were collected by using a triangular or rock dredge at approximately 80 m water depth at Liholmsrenna (Bergen, Norway) and were picked from coarse gravel, cobbles, and boulders. Spawning occurred spontaneously. Egg clutches were separated after fertilization and cultured in 50 ml plastic jars at 7 °C in Millipore-filtered and UV-treated seawater. Adults, embryos, and larvae were kept at 7 °C in the dark with exposure to light only during handling. Metamorphosis was induced in 18 dph old specimens by adding small rocks from the sampling area to metamorphically competent larvae. The offspring were cultured up to 31 dph (juvenile stage).

No special permissions were required to carry out sampling of adult specimens.

### Fixation and muscle staining

The animals were relaxed prior to fixation at 4 °C for 20 min by adding 3.2 % MgCl_2_ dropwise to the seawater. All specimens were fixed in 4 % paraformaldehyde in 0.1 M phosphate buffer (PB; pH = 7.3) for 1–3 h at room temperature (RT) or 4 °C. Subsequently, they were washed three times for 10–20 min in 0.1 M PB at RT or 4 °C and then stored in 0.1 M PB with 0.1 % NaN_3_ at 4 °C. All following steps were conducted at RT. Specimens were decalcified in 50 mM ethylene glycol tetraacetic acid (EGTA; pH = 7.3) for 2 h. EGTA solution was removed by washing the samples thrice for 10 min each in PB. All samples were then permeabilized for 1 h using 0.1 M PB with 2 % Triton X-100 (PBT; pH = 7.3). Staining of musculature was performed by fluorescence labeling of filamentous F-actin with Alexa Fluor 488 phalloidin (Invitrogen, Molecular Probes, Eugene, OR, USA) in a 1:40 dilution in PBT overnight (this and the subsequent steps were carried out in the dark). Afterwards, the samples were rinsed thrice for 10 min each in PB. Larvae were mounted in Fluoromount G (Southern Biotech, Birmingham, AL, USA) between two coverslips to allow scanning from both sides, whereas early juveniles and adults were mounted in PB on standard microscope slides. In total, 100 specimens of *Wirenia argentea* including early juveniles (and excluding adults) were examined to obtain a representative and comparable data set.

### Vibratome sectioning

Embedding, postfixation, and vibratome sectioning (Leica VT1200 S, Leica Microsystems, Wetzlar, Germany) of adult *Wirenia argentea*, as well as phalloidin staining of sections and all further preparations, followed the procedures described by Wollesen et al. [16].

### Microscopy and image processing

**Fig. 2:**
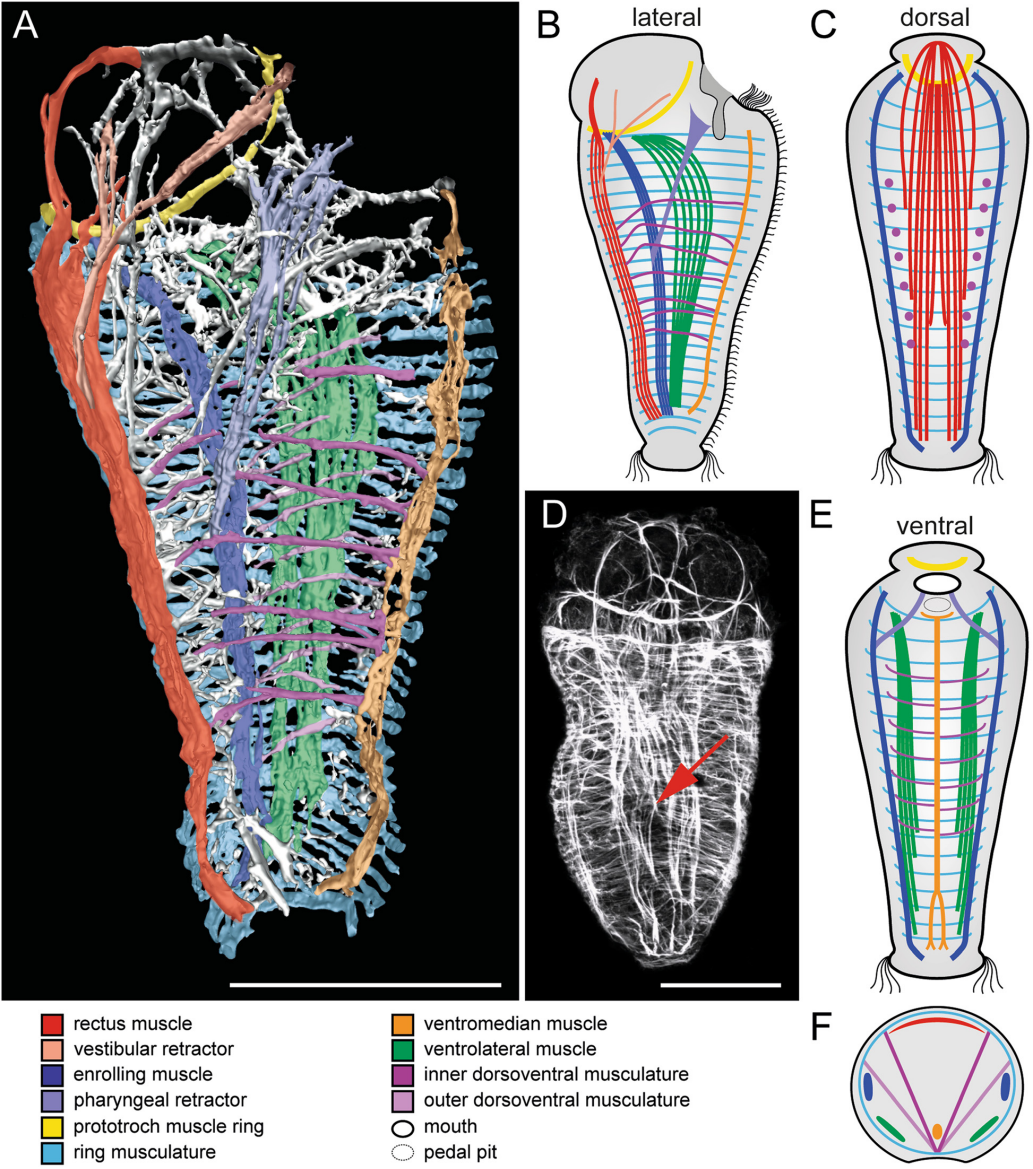
Myoanatomy of metamorphosing larvae of *Wirenia argentea* (Neomeniomorpha). Apical faces up in (**a**)-(**e**). For better visualization of individual muscular elements, the outer dorsoventral musculature is not depicted in panels (**b**)-(**e**) and only the ventral or dorsal half of the larva with section plane in the region of the enrolling muscle is shown in (**c**) and (**e**), respectively. Major muscle units are indicated by color code. **d** is a maximum intensity projection of a confocal image stack. **a** 3D reconstruction based on confocal microscopy data. Sagittal view shows left half of larval muscle system. All major muscle units are depicted. Note the seven-fold arrangement of the inner dorsoventral musculature and the multiple arrangement of outer dorsoventral musculature. **b** Schematic sagittal view shows left half of the larval musculature and corresponds to the 3D model in (**a**). **c** Schematic representation of the dorsal muscle system seen from ventral. **d** Scan in dorsal aspect. The red arrow indicates the point of bifurcation of the rectus muscle. **e** Schematic representation of the ventral muscle system seen from dorsal. **f** Schematic cross section indicates the relative position of individual muscular elements to each other. Scale bar: 50 μm

All samples were examined using a Leica TCS SP5 II confocal microscope (Leica Microsystems, Wetzlar, Germany). All larvae were scanned from two sides to allow for detailed comparison of all muscular structures. The software Leica Application Suite Advanced Fluorescence (LAS AF), Version 2.6.0 (Leica Microsystems, Wetzlar, Germany), was used to generate maximum intensity projections of confocal stacks of whole-mount preparations as well as Z-projections of confocal stack subsets to depict certain muscular structures. The 3D reconstruction (Fig. 2a) was compiled by using the isosurface rendering mode of the Imaris 7.3 Imaging Software (Bitplane, Zurich, Switzerland). Muscle-unspecific signal (from, e.g., spicule-secreting cells or cell borders) was removed prior to the isosurface rendering in order to allow for detailed visualization of the complex muscular system. Individual muscle system components were color-coded with 60 or 35 % opacity by using Adobe Illustrator CS5 (Adobe Systems, San José, CA, USA).

## Results

### Metamorphosis

Towards metamorphosis, all muscular components known from the late larva of *Wirenia argentea* (see [9] for details, also on early myogenesis) are well-developed and include the lateral enrolling muscles, paired ventrolateral muscles, a single ventromedian muscle, a prominent dorsal rectus muscle, seven pairs of dorsoventral muscles, and the body wall ring musculature (Fig. 2). In comparison to the late larva, the apical cap and the associated prototroch muscle ring are shifted dorsally, while their degeneration process also causes a reduction in diameter (Fig. 2a, b). The previously anteriorly fused enrolling muscle now terminates pairwise posterior to the prototroch muscle ring and the ventrolateral muscles reach until the most anteriorly positioned ring muscle (Fig. 2a-c, e). The split of the posterior end of the rectus muscle becomes more distinct (Fig. 2c, d). A set of outer dorsoventral muscles appears adjacent to the ring musculature and ventrolateral muscles and spans from ventromedian to lateral (Fig. 2a, b). The multiple arrangement of the outer dorsoventral musculature is not correlated to the seven-fold seriality of the inner dorsoventral musculature.

The anlagen of the paired vestibular retractor emerge from the rectus muscle and reach into the apical cap and towards the ventral mouth opening (Fig. 2a, b). The vestibule (or atrium), a cavity anterior to the mouth opening, has not yet developed. The paired pharyngeal retractor emerges from the enrolling muscle and attaches to the developing foregut (Fig. 2a, b, e).

### Early juvenile

**Fig. 3:**
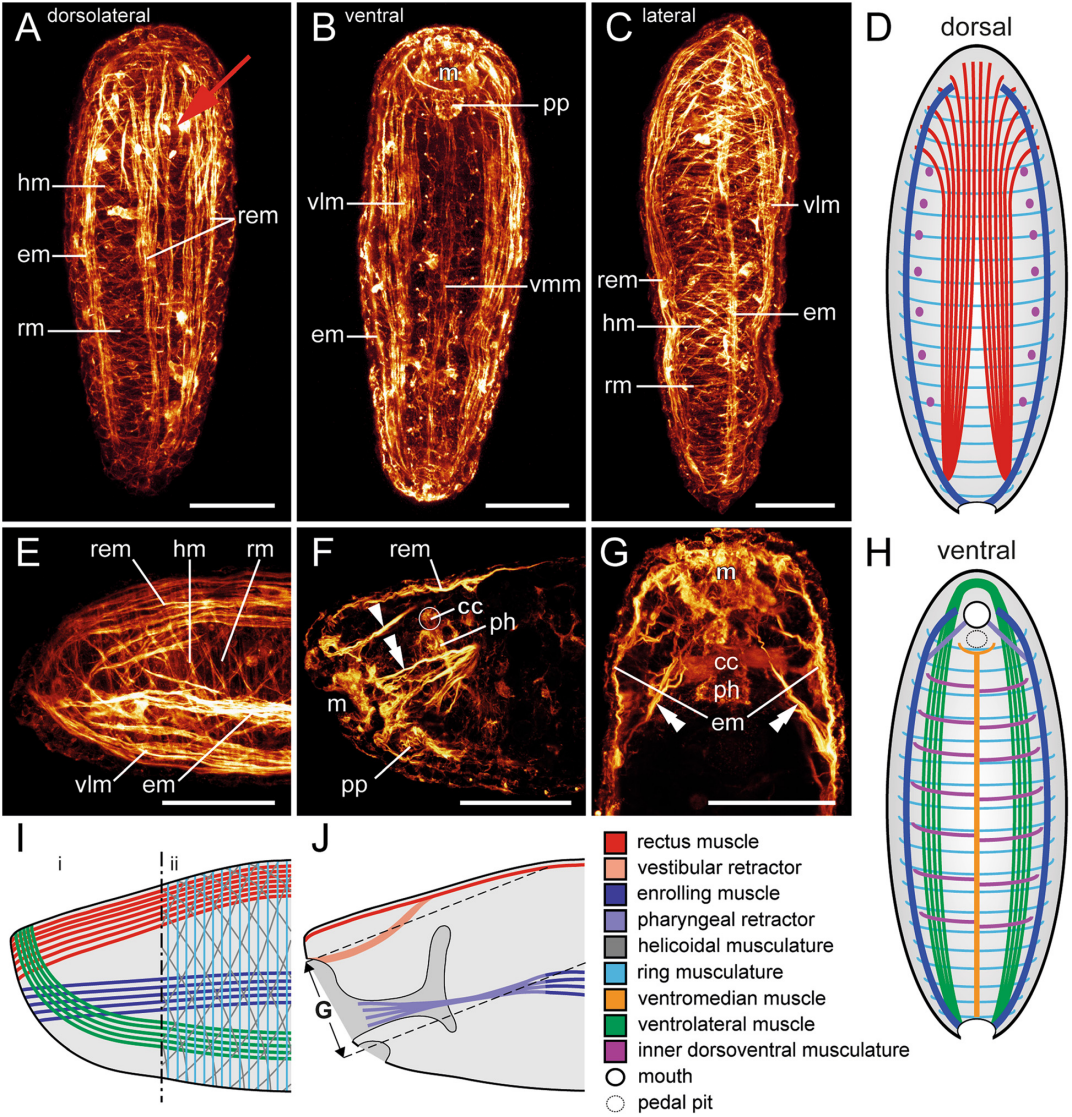
Myoanatomy of early juveniles of *Wirenia argentea* (Neomeniomorpha). Anterior faces up in (**a**)-(**d**), (**g**), (**h**). **a**-**c** and **e**-**g** are maximum intensity projections of confocal stacks or confocal stack subsets, respectively. For better visualization of individual muscular elements, the heliocoidal muscle layer and the outer dorsoventral muscles are omitted and only the ventral or dorsal half of the larva, with section plane in the region of the enrolling muscle, is shown in (**d**) and (**h**), respectively. Major muscle units in schematic representations are indicated by color code. **a** Dorsolateral aspect. The red arrow indicates the anteriorly shifted point of bifurcation of the rectus muscle. **b** Ventral aspect. **c** Right lateral aspect. **d** Dorsal muscle system seen from ventral. **e** Lateral view of the anterior region (left) showing the anterior-most arrangement of longitudinal muscle fibers. **f** Sagittal section of the anterior region (left) showing the vestibular retractor (arrowhead) and fibers of the terminally furcated pharyngeal retractor (double arrowhead). **g** Horizontal section of the anterior region shows the enrolling muscles (em) and the associated paired pharyngeal retractor (double arrowheads). **h** Ventral muscle system seen from dorsal. **i** Schematic representation of muscular elements of the anterior region (left). Scheme refers to panel (**e**). (i) without and (ii) with a schematic representation of the juvenile body wall musculature. **j** Sagittal section of the anterior region (left) with view on inner muscular elements. Scheme refers to (**f**). Dashed lines indicate the confocal image stack subset that was used in (**g**). Abbreviations: cerebral commissure (cc); enrolling muscle (em); helicoidal musculature (hm); mouth (m); pedal pit (pp); muscular pharynx (ph); rectus muscle (rem); ring musculature (rm); ventrolateral muscle (vlm); ventromedian muscle (vmm). Scale bars: 50 μm

After completion of metamorphosis, the larval apical cap and the associated prototroch muscle ring as well as the posterior telotroch have disappeared. The vermiform early juveniles still exhibit the same arrangement of distinct longitudinal larval muscle bundles, i.e., the paired enrolling and ventrolateral muscles, as well as the single ventromedian muscle and the rectus muscle (Fig. 3a-c, e). The fibers of the paired ventrolateral muscle fuse anteriorly, surrounding the vestibule, whereas the paired enrolling muscle and the rectus muscle terminate anteriorly at the vestibule margin (Fig. 3d, e, h, i). The bifurcation of the rectus muscle in early juveniles is more distinct and is situated more anteriorly than in the previous stage (Fig. 3a, d). All examined early juveniles still exhibit the larval condition of a seven-fold arrangement of the inner dorsoventral musculature (see [9]), confirming that their multiplication occurs later in development. The well-developed vestibular retractors are now correlated with the vestibule (Fig. 3f, j). The pharyngeal retractors, which split into several strands close to the insertion area, are attached to the developing pharynx (Fig. 3f, g, j). Oblique muscle fibers form an additional muscle layer between the outer ring muscles of the body wall musculature and the inner longitudinal muscle strands (Fig. 3a, c, e, i).

### Adult

**Fig 4:**
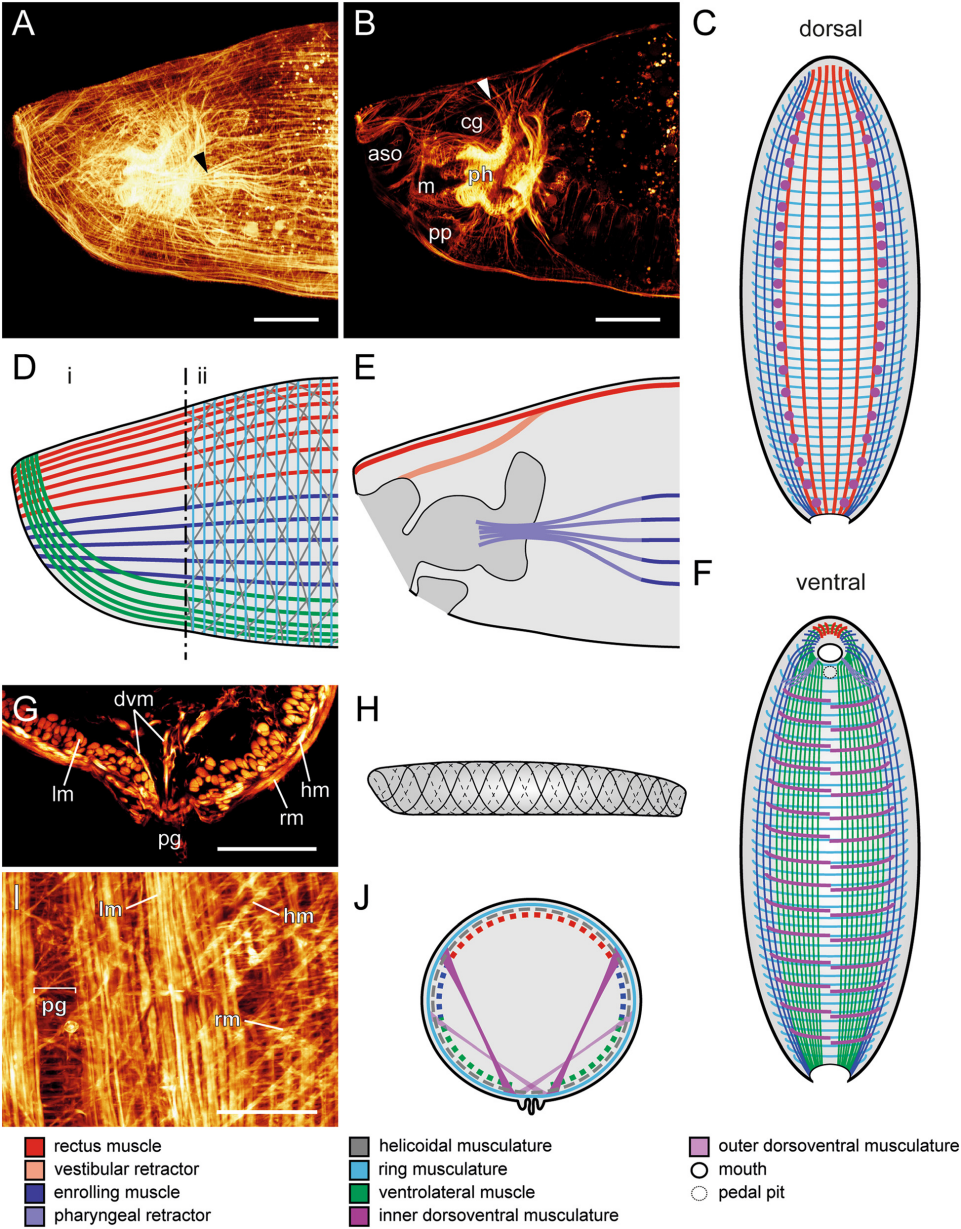
Adult myoanatomy of *Wirenia argentea* (Neomeniomorpha). Anterior is to the left in (**a**), (**b**), (**d**), (**e**), (**h**) and up in (**c**), (**f**). (**a**), (**b**), (**g**), (**i**) are maximum intensity projections of confocal stacks or confocal stack subsets, respectively. For better visualization of individual muscular elements, the helicoidal muscle layer and the set of outer dorsoventral musculature are omitted and only the ventral or dorsal half of the animal with section plane in the region of the enrolling muscle is shown in (**c**) and (**f**), respectively. Major muscle units in schematic representations are indicated by color code. Cross section in (**g**) was generated by vibratome sectioning. **a** Lateral view of the anterior region with identical arrangement of longitudinal muscle fibers as in juveniles. The diverging pharyngeal retractor is indicated by the black arrowhead. **b** Inner muscular organization of the anterior region with vestibular retractor (white arrowhead). **c** Dorsal muscle system seen from ventral. **d** Schematic view of muscular elements of the anterior region. Scheme refers to (**a**) and indicates the anterior-most arrangement of longitudinal muscle fibers and their correlation to the larval longitudinal muscles in (i) and the three-layered body wall musculature in (ii). **e** Sagittal section of the anterior region and view on inner muscular elements. Scheme refers to (**a**) and (**b**). **f** Ventral muscle system seen from dorsal. (**g**) Cross section through the ventral body. **h** Schematic representation of the arrangement of the intermediate helicoidal muscle layer; lateral view. **i** Detail of a ventral view. **j** Schematic cross section in the mid-body region. Abbreviations: atrial sense organ (aso); cerebral ganglion (cg); fibers of outer or inner dorsoventral musculature (dvm); helicoidal musculature (hm); longitudinal musculature (lm); mouth (m); pedal groove (pg); pedal pit (pp); muscular pharynx (ph); ring musculature (rm). Scale bars: 50 μm

The myoanatomy of the considerably elongated adult *Wirenia argentea* is rather simple compared to the larva. Instead of distinct longitudinal muscle bundles, the adults exhibit a homogeneously distributed layer of longitudinal muscle fibers, which is interrupted and slightly thickened adjacent to the pedal groove (Fig. 4a, c, d, f, g, i, j). These longitudinal fibers form the innermost layer of the three-layered adult body wall musculature (Fig. 4g, j). Both, the outer ring and the intermediate oblique musculature, form two continuous layers that cross the pedal groove proximally (Fig. 4i). The often-mentioned oblique or diagonal muscle fibers (e.g., [13, 17, 18]) are in fact helicoidally arranged and we thus term them “helicoidal musculature” (Fig. 4g, h, i, j). The antidromically arranged muscle fibers of the helicoidal musculature form a meshwork with rhomboid texture. Two serially arranged dorsoventral muscle sets can be distinguished, an inner and an outer set, in which each set has an alternated arrangement of its individual components. The outer dorsoventral muscles span from lateral to ventral and intercross dorsally of the pedal groove (Fig. 4j). This is in contrast to the inner dorsoventral muscles, which span from dorsolateral to ventral without intercrossing (Fig. 4j). Both systems generate a superficial impression of pseudo-segmentation by narrowing the midgut in more or less regular intervals. The anterior region still exhibits the dorsally differentiated paired vestibular retractors, which are attached to the vestibule (Fig. 4b, e). The vestibule encloses the atrial sense organ and the mouth opening (Fig. 4b). The paired pharyngeal retractors appear to be formed by concentration of medially segregated longitudinal fibers of the body wall musculature. They split and attach to the muscular pharynx in the region of the radula (Fig. 4a, e).

### Polyplacophoran myogenesis

**Fig. 5:**
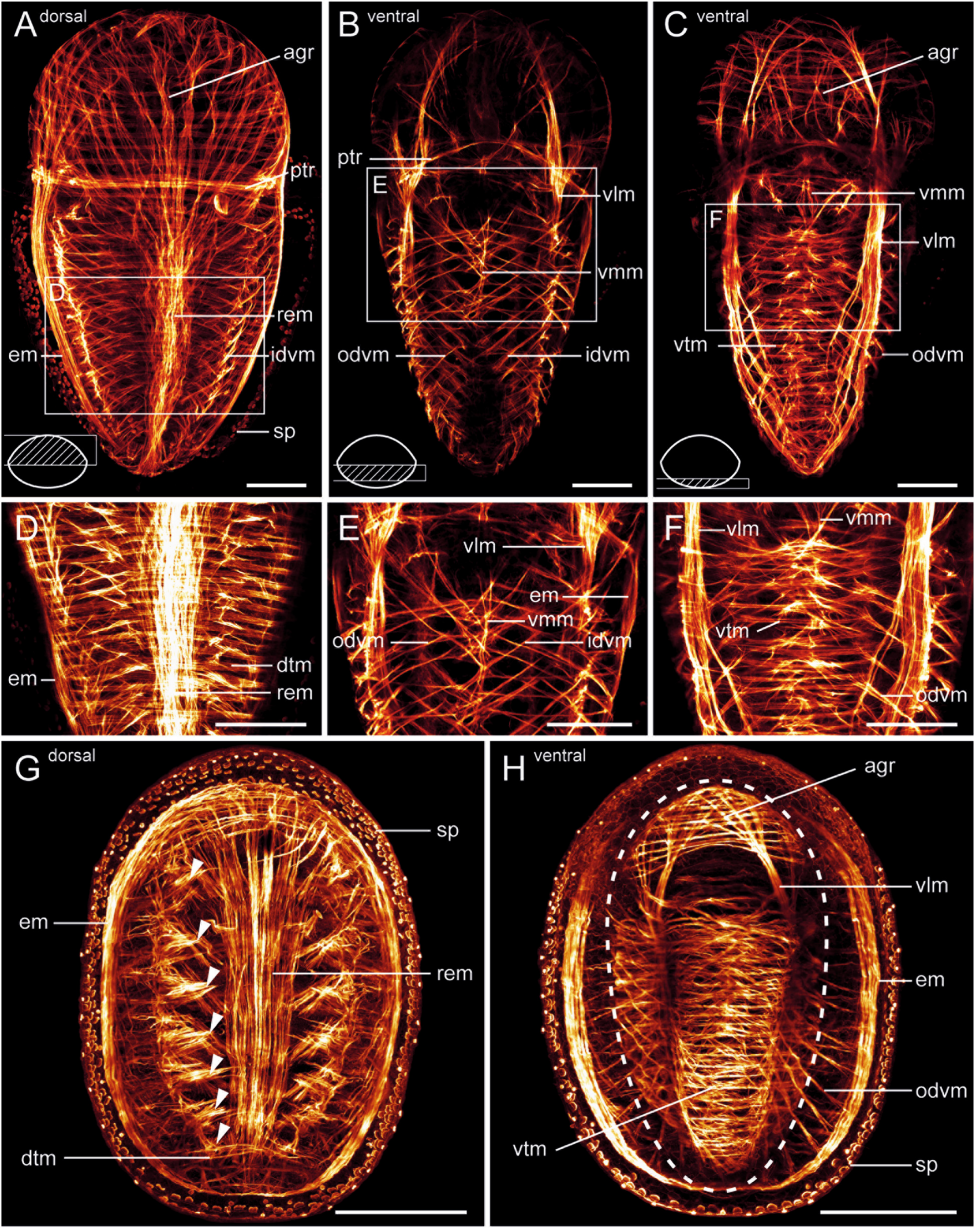
Late larval and early juvenile myoanatomy of *Leptochiton asellus* (Polyplacophora). Apical/anterior faces up in all panels. All panels are maximum intensity projections of confocal image stack subsets. Hatched area of the simplified schematic cross sections in the lower left corner of (**a**)-(**c**) indicates the processed confocal image stack subset. Age of depicted specimens is 15 days post hatching (dph) in (**a**)-(**c**) and 31 dph in (**g**) and (**h**). **a** Dorsal musculature of a late larva. The pretrochal musculature forms an apical grid (agr). Dorsal insertion sites of the multiple inner dorsoventral muscles (idvm) are clearly visible. **b** Part of the ventral musculature of a late larva. **c** Ventral-most musculature of a late larva. **d** Detail of (**a**). **e** Detail of (**b**). **f** Detail of (**c**). **g** Dorsal musculature of an early juvenile. Note the condensed seven muscle strands of the inner dorsoventral musculature (arrowheads), which are associated with seven juvenile shell plates. **h** Ventral musculature of an early juvenile. Mantle groove is indicated by dashed line. Note the ventral vestiges of the larval apical grid (agr) in the anterior region. Ventral transversal musculature (vtm) as well as ventrolateral muscles (vlm) are still present in the foot. Abbreviations: apical grid (agr); dorsal transversal musculature (dtm); enrolling muscle (em); inner dorsoventral musculature (idvm); outer dorsoventral musculature (odvm); prototroch muscle ring (ptr); rectus muscle (rem); spicule-secreting cells (sp); ventrolateral muscle (vlm); ventromedian muscle (vmm); ventral transversal musculature (vtm). Scale bars: 50 μm in (**a**)-(**f**) and 100 μm in (**g**) and (**h**)

The larval muscle system of the polyplacophoran *Leptochiton asellus* largely resembles that of *Wirenia argentea* and contains a paired enrolling muscle, a paired ventrolateral muscle, a single ventromedian muscle, a prominent dorsal rectus muscle, and a prototroch muscle ring (Fig. 5a-c, e). The apical grid of the pretrochal episphere consists of outer ring musculature and inner longitudinal fibers of the anteriorly diverged paired ventrolateral and enrolling muscle as well as the rectus muscle (Fig. 5a, c). The posttrochal hyposphere lacks such a ring musculature but comprises a ventral and a dorsal transversal musculature which covers the dorsal rectus muscle (Fig. 5d, f). Furthermore, two sets of multiple serially arranged dorsoventral muscles, an outer and an inner set, can be distinguished in the larval muscle system.

In early juveniles of *Leptochiton asellus*, after completion of metamorphosis, the prototroch with the associated prototroch muscle ring and the larval episphere have disappeared. Nevertheless, the ventral part of the apical grid is retained for some time (Fig. 5g, h). The juvenile foot still exhibits the ventral transversal musculature and the paired ventrolateral muscle, but lacks the ventromedian muscle (Fig. 5h). The juvenile enrolling muscle has already the circular adult appearance (Fig. 5g, h). The inner dorsoventral muscles are dorsally concentrated into seven paired muscle bundles which correspond to the developing seven dorsal shell plates of the juvenile (Fig. 5g), whereas the outer dorsoventral muscles are still serially arranged (Fig. 5h).

## Discussion

**Fig. 6:**
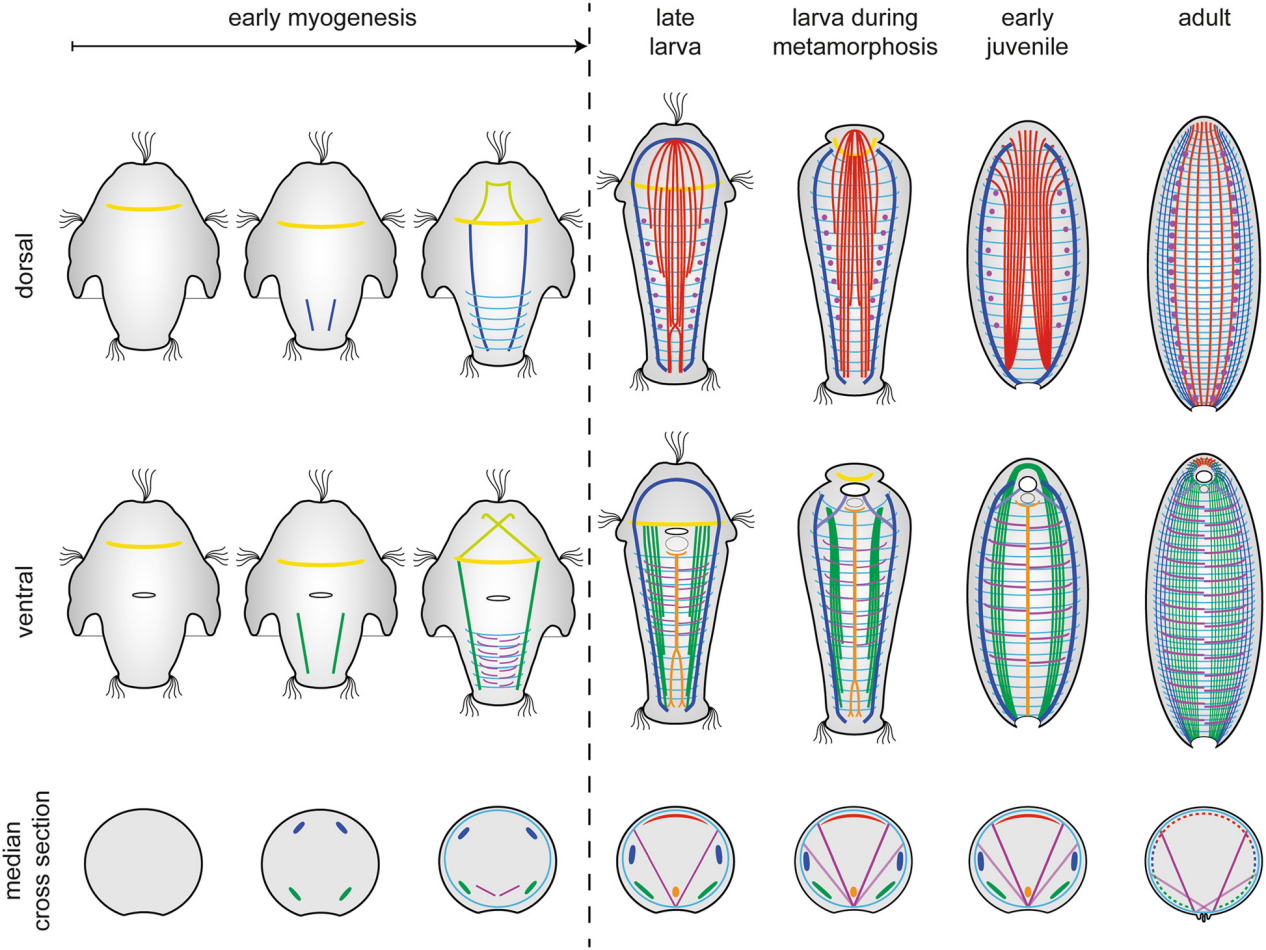
Schematic summary of myogenesis in *Wirenia argentea* (Neomeniomorpha). The muscular system of each stage is shown in dorsal and ventral view. Dorsal muscle systems are seen from ventral, ventral muscle systems are seen from dorsal. Median cross sections indicate the relative position of each muscular element. For better visualization of individual muscular elements, the outer set of the dorsoventral musculature is not shown in ventral and dorsal schemes, and the heliocoidal muscle layer is omitted in all aspects. Muscle units are indicated by color code: yellow, prototroch muscle ring; dark blue, enrolling muscle; light blue, pharyngeal retractor; green, ventrolateral muscle; light green, pretrochal muscle system; violet, inner dorsoventral musculature; light violet, outer dorsoventral musculature; cyan, ring musculature; red, rectus muscle; orange, ventromedian muscle. For details on early myogenesis, see [9]

**Fig. 7:**
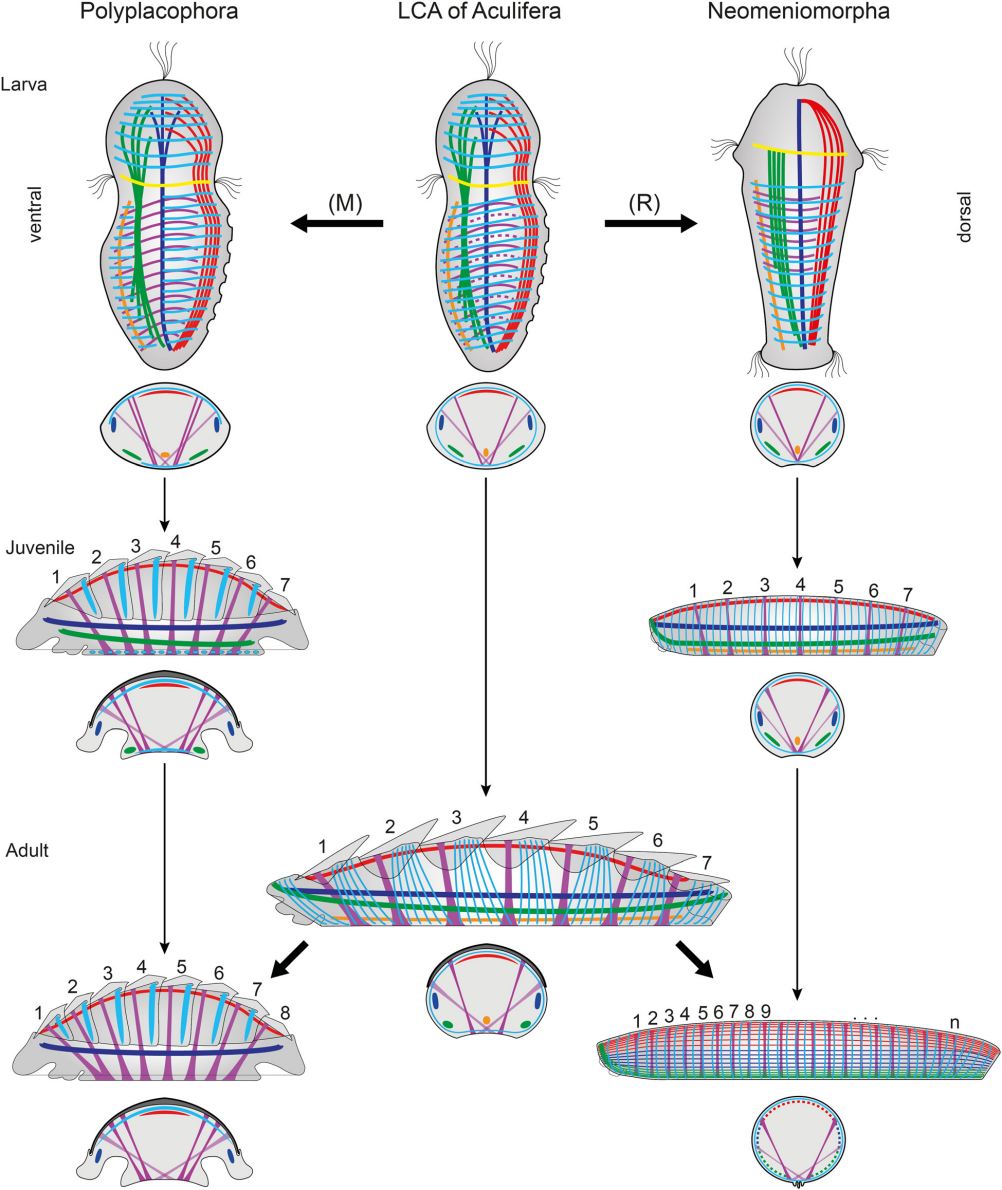
Reconstruction of the larval and adult myogenic body plan of the last common aculiferan ancestor. Apical is up in all larval schemes. Anterior is to the left in schemes of juveniles as well as adults. Schematic illustrations indicate ontogeny (fine arrows) from late larval until the adult stage. Evolutionary transitions are indicated by massive arrows. Because it is not determinable whether the larval stages of the last common ancestor exhibited a neomeniomorph-like seven-fold seriality or a polyplacophoran-like multiple seriality of inner dorsoventral musculature, the additional muscles are stippled. Referring to this, the (R) indicates a possible numeric reduction of inner dorsoventral musculature during neomeniomorph evolution and, as alternative, the (M) indicates a possible multiplication of inner dorsoventral musculature during polyplacophoran evolution. All stages are shown translucent in lateral view from the left with an additional scheme of a median cross section below. Serial arrangement of shell plates and the corresponding dorsoventral musculature is indicated by numbering of the depicted structures. Note the shared seven-fold seriality in juveniles of Polyplacophora and Neomeniomorpha as represented by dorsoventral muscles and shell plates. For better visualization, the outer dorsoventral musculature is exclusively drawn in cross section schemes, and helicoidal musculature is not depicted. Homologous muscular elements are indicated by color code corresponding to previous figures: yellow, prototroch muscle ring; dark blue, enrolling muscle; green, ventrolateral muscle; violet, inner dorsoventral musculature; light violet, outer dorsoventral musculature; cyan, ring musculature/transversal musculature; red, rectus muscle; orange, ventromedian muscle

So far, it had not been possible to reliably homologize muscular subsets between the greatly diverging muscular body plans of adult Neomeniomorpha and Polyplacophora. The similarities in the neomeniomorph and polyplacophoran larval myoanatomy, in contrast, as well as the postmetamorphic muscular remodeling in *Wirenia argentea*, enable inferences on homologous structures in both taxa. The complex larval muscle system of neomeniomorphs and polyplacophorans suggests a common ancestry as well as homology of the involved muscle sets [9]. Accordingly, larvae of both molluscan representatives possess homologous longitudinal muscles including a dorsal rectus muscle, a paired lateral enrolling muscle, a paired ventrolateral muscle, and a ventromedian muscle. In addition, they share a prototroch muscle ring as well as outer and inner dorsoventral musculature. However, the two larval types also show significant differences: neomeniomorph larvae exhibit a subepidermal ring musculature in the posttrochal trunk region, whereas the polyplacophoran larvae exhibit a subepidermal posttrochal dorsal and ventral transversal musculature, as well as a pretrochal apical grid, which consists of subepidermal ring musculature and inner longitudinal muscle fibers (Figs. 6 and 7).

Neomeniomorpha and Polyplacophora differ fundamentally in their adult myoanatomy. The worm-shaped Neomeniomorpha possess a multitude of outer and inner dorsoventral muscles and a rather simple three-layered body wall musculature, which consists of a more or less homogeneously distributed inner layer of longitudinal fibers, an intermediate helicoidal layer, and an outer ring muscle layer. In contrast, the myoanatomy of adult Polyplacophora is highly complex [19]. Several larval features such as the rectus muscle, the enrolling muscle, the dorsal transversal muscles, and the dorsoventral muscles are still present, but are transformed and adapted to the adult habitus. Thus, the adults exhibit an eight-fold serial arrangement of outer and inner dorsoventral muscles, which are associated with the eight dorsal shell plates, and a circular enrolling muscle (Fig. 7). Other larval features such as the apical grid, the prototroch muscle ring, the ventromedian muscle, the paired ventrolateral muscle, and the ventral transversal musculature are transient larval structures that are absent in adult polyplacophorans.

### From polyplacophoran- to worm-like: remodeling of the larval musculature in Neomeniomorpha

#### Transitory elements

A transitory prototroch muscle ring is common throughout Mollusca and is known from the Polyplacophora [14], Gastropoda [20], Bivalvia [21, 22], as well as Caudofoveata [23] (but is lacking in Scaphopoda – see [24]) and is herein confirmed for the neomeniomorph *Wirenia argentea* [9] (Fig. 2). This muscle ring and the associated prototroch are lost during metamorphosis (Table 1). Because such a circular muscle underlying a ciliary band is also found in, e.g., annelids [25, 26], nemerteans [27], and entoprocts [28], it most likely constitutes a molluscan plesiomorphy.

**Table 1:** Fate of individual larval muscles in *Wirenia argentea* (Neomeniomorpha)

	ᅟ

The characteristic anterior fiber arrangement of the longitudinal muscle layer of adult Neomeniomorpha reveals the developmental fate of the distinct larval (and juvenile) longitudinal muscle units (compare Figs. 3 and 4). The recovery of the same fiber arrangement in both early juveniles and adults clearly argues for muscular transformation by two-dimensional expansion of the originally distinct rectus, enrolling, and ventrolateral muscle bundles into a secondary uniform layer, rather than for reduction and *de novo* genesis of the longitudinal portion of the adult body wall musculature. Accordingly, the larval rectus, enrolling, and ventrolateral muscles are transformed during metamorphosis and form the innermost layer of the adult body wall musculature. Together with the larval ring and helicoidal musculature, these formerly distinct longitudinal larval muscle bundles form the characteristic adult three-layered body wall musculature. Transitory elements such as the ventromedian muscle and the prototroch muscle ring are lost during neomeniomorph metamorphosis (indicated by “absent”). The number of individual muscles of the inner and outer sets of dorsoventral musculature increases during postmetamorphic body elongation. The larval (and early juvenile) seven-fold seriality of the inner dorsoventral muscles is thus not present in adult *Wirenia argentea*

Comparative analysis of polyplacophoran and neomeniomorph myogenesis reveals the existence of a single ventromedian muscle with a posterior bifurcation in both clades [9]. Specimens of *Wirenia argentea* still possess this transient character as early juveniles and show a total loss as adults (Table 1), whereas in the polyplacophoran *Leptochiton asellus* this muscle is already reduced during metamorphosis. Based on the same relative position and orientation as well as the shared characteristic of a posterior bifurcation, the transient ventromedian muscles in Neomeniomorpha and Polyplacophora are considered homologous [9]. A similar longitudinal ventromedian muscle is found in annelids and has recently been suggested to be homologous to the ventromedian muscle of neomeniomorphs and polyplacophorans, as well as to ventral muscles of several other protostome taxa [29] (but see [30] for critical discussion). In case this will stand critical future assessment, the ventromedian muscle would likewise constitute a plesiomorphic character for Mollusca.

#### Rectus muscle

During subsequent development, the posterior bifurcation of the larval rectus muscle becomes more distinct in metamorphosing larvae and reaches an appearance with an anterior divergence in the early juveniles. This mode of development is similar to that in polyplacophoran larvae [14]. In metamorphosing *Wirenia* larvae and early juveniles the vestibular retractors originate from the rectus muscle and this association is permanent. Also in adult *Wirenia argentea* the vestibular retractors attach dorsally, but they are associated with the dorsal portion of the longitudinal layer of the body wall musculature. Thus, this portion of the longitudinal body wall musculature is here interpreted as a derivative of the larval rectus muscle, formed by expansion and dissociation of this formerly distinct muscle bundle (Fig. 6; Table 1).

#### Enrolling muscle

Neomeniomorph metamorphosis is characterized by a continuous elongation of the posterior trunk and a gradual degeneration of the apical cap. This degeneration leads to a degradation of the former apical interconnection of the larval enrolling muscle. The result is a pairwise occurrence of the lateral enrolling muscle in metamorphosing larvae. This larval enrolling muscle persists through metamorphosis and is still present in early juveniles, where it terminates anteriorly at the vestibule margin. Additionally, in both developmental stages, it is always associated with a paired pharyngeal retractor. This muscle originates from the enrolling muscle and runs anteriorly to the developing foregut in metamorphosing larvae. Early juveniles show the insertion of the furcated terminal ends of the pharyngeal retractor muscles to the muscular pharynx region. A comparison of this situation with the adult myoanatomy shows that the terminal diverging fraction of the pharyngeal retractors in early juveniles represents most probably the anlagen of the robust retractor muscles that are restricted to the radula region of adult *Wirenia argentea* [31]. These retractor muscles are, however, attached to the lateral portion of the longitudinal muscle layer of the adult body wall musculature. This particular association argues for a derivation of the lateral portion of the longitudinal muscle layer from the larval enrolling muscle. This is supported by the identical anterior insertion of the enrolling muscle in early juveniles and the lateral portion of the longitudinal muscle layer in adult *W. argentea*. Consequently, the lateral portion of the adult longitudinal muscle layer is formed by transformation and two-dimensional expansion of the formerly distinct larval (and juvenile) enrolling muscle (Table 1).

#### Ventrolateral muscle

As in polyplacophorans (cf. [14]; this study), the paired ventrolateral muscle in *Wirenia argentea* persists after metamorphosis and is still present in early juveniles. In this stage the fibers of the paired muscle are fused anteriorly and surround the developing vestibule. This identical anterior fiber arrangement is still recognizable in the most ventral part of the longitudinal muscle layer in adult *W. argentea*, and thus we consider this part as most likely derived from the distinct larval (respectively juvenile) paired ventrolateral muscle.

#### Helicoidal musculature

The helicoidal muscle fibers appear first in the worm-shaped early juveniles of *Wirenia argentea*, where they form an obliquely arranged meshwork outside the longitudinal larval muscles. This meshwork is condensed in adults and forms the continuous intermediate layer of helicoidal muscle fibers of the body wall musculature (Table 1). This layer was formerly described in neomeniomorphs as consisting of oblique muscle fibers; in *W. argentea*, however, we found this layer to be formed by helicoidal musculature, a feature of the body wall that may be unique for Neomeniomorpha.

#### Ring and transversal musculature

In *Wirenia argentea*, the subepidermal ring muscle layer appears early during ontogeny and is restricted to the posterior trunk region. Initially, it covers the longitudinal larval muscle strands without an intermediate helicoidal muscle layer in between. After completion of metamorphosis, it overlies the emerging helicoidal musculature and represents the outermost muscle layer of the body wall musculature in early juveniles and adults (Table 1).

Ring musculature is present in (larvae of) all three aculiferan subgroups. As in *W. argentea*, the ring musculature of the chaetodermomorph *Chaetoderma nitidulum* appears early during ontogeny and is also restricted to the posttrochal larval trunk region [23]. Therefore, and because adult Chaetodermomorpha also possess a three-layered body wall musculature with outer ring muscles, we consider the chaetodermomorph and neomeniomorph ring musculature as homologous. However, the ring musculature of polyplacophorans is restricted to the transient larval apical grid, which was considered a rudiment of an ancestral aplacophoran-like body wall musculature [14]. Similar to the situation in Neomeniomorpha, the polyplacophoran larval ring musculature overlies longitudinal muscle fibers, which derive from the larval rectus, enrolling, and ventrolateral muscles. Thus, the polyplacophoran larval ring musculature is most likely homologous to the aplacophoran ring musculature. The common possession of homologous subepidermal (larval) ring musculature in Chaetodermomorpha, Neomeniomorpha, and Polyplacophora implies that it was already present in the last common aculiferan ancestor.

In contrast to the aplacophoran larvae, the posttrochal region of polyplacophoran larvae is devoid of ring muscles, but does exhibit dorsal and ventral transversal musculature. After completion of metamorphosis the dorsal transversal musculature is initially condensed into six, later into seven bundles that underlie the overlapping shell plates in early juveniles and adults in the region of the apophyses (Fig. 7). The evenly arranged ventral transversal musculature is still present in the juvenile foot, but is absent in adults (Fig. 7). Even before the discovery of the ventral transversal musculature, the (dorsal) transversal musculature was considered to be derived from an ancestral ring musculature and, thus, to be homologous to the aplacophoran ring musculature [18]. The discovery of the ventral counterpart significantly increases the probability of homology, and, accordingly, we consider the dorsal and ventral transversal musculature of polyplacophorans homologous to the aplacophoran ring musculature and thus as rudiments of an ancestral continuous ring muscle layer (Fig. 7).

#### Dorsoventral musculature

The inner dorsoventral muscles arise simultaneously during early *Wirenia argentea* development and retain a seven-fold seriality throughout metamorphosis, whereas the outer dorsoventral muscles appear later, i.e., in metamorphosing larvae, and are not linked to a seven-fold seriality. Instead, they show a multiple serial arrangement similar to the dorsoventral musculature in early stages of polyplacophoran myogenesis (cf. [14]). Since this situation is still present in early juveniles of *W. argentea*, the multiplication into a series of numerous inner dorsoventral muscles is clearly a postmetamorphic event and occurs during the transition towards the adult stage (Table 1).

Since the formation of the eight-fold seriality of the dorsoventral musculature in extant single-shelled monoplacophorans was traditionally considered a recapitulative element and as such a link to the eight shell plates-bearing polyplacophorans (e.g., [32, 33]), the reconstruction of the ancestral condition in Aculifera is of great significance also for inferences concerning conchiferan evolution. Thus, the recurrent discovery of a seven-fold seriality of dorsoventral musculature and the associated dorsal hard parts in ontogenetic stages and adults as well as in the fossil record of aculiferan taxa (e.g., the Silurian seven-shelled aplacophoran *Kulindroplax*; see [7]) are remarkable and may hint towards such a condition also in the last common ancestor of Conchifera. Since a reliable conchiferan phylogeny is not yet at hand and monoplacophoran ontogeny remains unknown, this issue cannot be finally settled at present [9].

The unknown ancestral conchiferan condition poses a serious problem not only for reconstructing the molluscan ancestor, but also complicates the determination of ancestral aculiferan conditions by impeding outgroup comparison. Thus, the presence of multiple serially arranged inner dorsoventral musculature in polyplacophoran larvae, on the one hand, and the seven-fold seriality of inner dorsoventral musculature in neomeniomorph larvae, on the other hand, raises an obvious issue concerning the ancestral aculiferan condition. In this case the principle of parsimony offers two scenarios with equal probability: 1.) The polyplacophoran-like multiple serial arrangement represents the primary condition in aculiferan larvae. This scenario implies that the multiple serially arranged stage during the development of the inner dorsoventral musculature was reduced to a seven-fold seriality in the neomeniomorph lineage (Fig. 7). 2.) The neomeniomorph-like seven-fold arrangement of the inner dorsoventral musculature represents the primary condition in aculiferan larvae. Consequently, the multiple serial arrangement in polyplacophoran larvae and the subsequent concentration into seven pairs of inner dorsoventral muscles would be interpreted as novel feature in the polyplacophoran lineage (Fig. 7, upper row). Regardless of this, however, both Neomeniomorpha and Polyplacophora recapitulate a seven-fold seriality of inner dorsoventral musculature, which arises synchronously (albeit from condensation of multiple fibers in polyplacophorans while they form as individual myocytes in neomeniomorphs) in their respective early juveniles, which is why we consider such a seven-fold muscular seriality the ancestral state for adult Aculifera (Fig. 7).

### Aplacophoran body wall musculature: ancestral or derived condition?

A three-layered body wall musculature is widespread among worm-like lophotrochozoans and was thus likely a character of their last common ancestor [3, 14, 34–36]. Several vermiform phyla including Annelida, Platyhelminthes or Nemertea exhibit – at least in certain lineages – such a three-layered body wall musculature (e.g., [37–39]), similar to that in Neomeniomorpha and Chaetodermomorpha. Hence, the existence of such a three-layered body wall musculature in Aplacophora was considered plesiomorphic for Mollusca [13, 14, 40] and the polyplacophoran enrolling muscle as well as the rectus muscle and the larval apical grid were regarded as derivatives of an aplacophoran-like body wall musculature [13, 14, 41]. A thickened ventrolateral part of the inner longitudinal layer is often found in the adult aplacophoran body wall musculature and was traditionally considered homologous to the polyplacophoran enrolling muscle [13, 33, 42, 43] (but see [3, 40] for different view).

The muscular remodeling and the perpetuation of the identical anterior arrangement of longitudinal muscle fibers strongly suggest that the so-called enrolling muscle of adult Neomeniomorpha is not homologous to the polyplacophoran enrolling muscle, but, instead, to the ventrolateral muscle found in polyplacophoran larvae and juveniles. The ring muscle in the foot of Monoplacophora has also been proposed to be homologous to the enrolling muscle in Polyplacophora and Aplacophora [13, 33], but differences in their position and innervation patterns have been used as counter arguments of homology [40]. To eventually resolve this issue, data on monoplacophoran myogenesis are of crucial importance.

The paired appearance of the larval enrolling muscle in Neomeniomorpha and Polyplacophora, as well as its subsequent transformation by anterior and posterior fusion during polyplacophoran metamorphosis into the adult circular enrolling muscle, implicates that this pairwise occurrence might be interpreted as a plesiomorphic character (Fig. 7). Following this line of reasoning, the rectus muscle and paired ventrolateral muscle in neomeniomorph and polyplacophoran larvae might be interpreted as plesiomorphic characters as well (Fig. 7).

The vast plasticity of myoanatomy and myogenesis of extant conchiferan taxa, as well as the lack of ontogenetic data for Monoplacophora, precludes detailed assumptions about the myogenic bauplan of the last common ancestor of Conchifera. However, it was shown that a vermiform body and an associated three-layered body wall musculature evolved several times independently within certain conchiferan lineages from a much more complex ancestral system in, e.g., bivalves (shipworms) as well as in various gastropods [3, 40, 44–46]. In contrast to the above-mentioned earlier views, the Neomeniomorpha can now be added to the list of secondarily “simplified” (or, rather, “vermified”) mollusks. The multiple evolutionary origins of worm-shaped mollusks and the primary absence of a three-layered body wall musculature in the Conchifera, as well as the data presented here on myogenesis in the Neomeniomorpha, are indications that this character was supposedly not part of the ancestral molluscan bauplan. Accordingly, the three-layered body wall musculature in (aplacophoran) mollusks is not homologous to that of any other spiralian taxon.

## Conclusions

The developmental and paleontological data ([7, 9]; this study) currently available suggest that the body of the aculiferan stem species most likely had a seven-fold arrangement of inner dorsoventral musculature and was covered dorsally by seven corresponding shell plates (Fig. 7). The myoanatomy was complex and was composed of subepidermal ring musculature and distinct longitudinal strands of a laterally situated paired enrolling muscle, a paired ventrolateral muscle, a single ventromedian muscle, and a dorsal rectus muscle (Fig. 7). Subsequent body elongation in the aplacophoran stem lineage required a secondary multiplication of dorsoventral musculature, and the loss of dorsal exoskeletal elements (shell plates) fueled the secondary formation of a three-layered body wall musculature as exhibited by recent aplacophorans. The ontogeny of the musculature of the neomeniomorph *Wirenia argentea*, which involves postmetamorphic multiplication of the dorsoventral musculature as well as transformation of the complex larval muscular conditions into the simple adult body wall musculature (Fig. 6), is thus another prime example of ontogenetic recapitulation of evolutionary history and provides a window into deep molluscan evolution. The unexpected finding that a once-thought basal character complex is, in fact, highly derived and secondarily simplified, dramatically highlights the importance of ontogenetic data for inferences concerning animal evolution (see also [48]).

### Ethics approval

The authors confirm that all experiments conducted in this study comply with institutional, national, or international guidelines.
